# Editorial: Autoantibodies and autoimmune neuromuscular disorders

**DOI:** 10.3389/fneur.2025.1687550

**Published:** 2025-09-19

**Authors:** Pritikanta Paul, Qihua Fan

**Affiliations:** ^1^Department of Neurology, University of California, San Francisco, San Francisco, CA, United States; ^2^Department of Neurology, Virginia Commonwealth University, Richmond, VA, United States

**Keywords:** neuromuscular medicine, autoimmune neuromuscular diseases, myasthenia (myasthenia gravis—MG), autoantibodies, immunotherapies

Neuromuscular medicine is being propelled by a deeper understanding of the immune system's intricate role in pathogenesis of autoimmune neuromuscular disorders. This Research Topic of articles on “*Autoantibodies and autoimmune neuromuscular disorders*” underscores this progress and advances our knowledge from disease mechanisms to emerging therapeutic strategies.

The articles collectively demonstrate that autoantibodies are more than just diagnostic markers; they are dynamic indicators that can guide treatment and predict outcomes. The case report by Appeltshauser et al. on a patient with autoimmune nodopathy (AN) emphasizes AN as a distinct entity with unique pathobiology in the approach to atypical “GBS/CIDP-like” neuropathies. A key insight from this report is that pathogenic antibodies can rarely shift their targets, profoundly influencing clinical course and treatment responsiveness. Clinicians should recognize specific phenotypes for nodal/paranodal antibodies (e.g., contactin-1, Caspr1, NF155/NF186) associated neuropathies. Furthermore, biomarkers such as antibody titers and serum neurofilament light (sNFL) offer critical insights into disease activity, and screening for renal involvement is essential when contactin-1 is implicated. Ultimately, this case validates AN as distinct from classic demyelination and underscores the importance of precision immunotherapy and longitudinal immunophenotyping.

The original research study by Fontana et al. demonstrates applicability of a multi-specific line blot assay, which detected myositis-specific and myositis-associated autoantibodies (MSA and MAA) in 74% of patient sera, with a 64.8% concordance rate with the final diagnosis. In contrast to traditional diagnosis of inflammatory myopathy by clinical features and muscle biopsy, this method allows for a potentially less invasive and more precise diagnostic workflow.

Zhang Q. et al. explores a novel therapeutic direction by investigating the role of natural killer (NK) cells in myasthenia gravis (MG). The researchers found that peripheral NK cells in MG patients with more severe disease are characterized by a loss of total NK cells and impaired cytotoxic activity. They identified an underlying regulatory mechanism involving the IL-6/IL-21 pathway, which induces the NK cell exhausted signature. Crucially, the study suggests that inhibiting this pathway and restoring NK cell function could represent a new therapeutic avenue for MG. This exploration of cellular and molecular mechanisms of autoimmunity makes a case for considering immunomodulatory strategies that go beyond B-cell and T-cell targeting. NK cell pathway dysregulation is increasingly recognized in autoimmune and neuroimmunological diseases (1). While NK cells exhibit context-dependent pathogenic or regulatory roles, their biomarker potential and therapeutic applications are actively being explored. Emerging strategies include cytokine-based activation (e.g., IL-2, IL-15 superagonists), adoptive NK cell therapies, and modulation of NK checkpoints, with several approaches in early clinical development.

Moving from foundational research to clinical application, two original articles focus on the efficacy of innovative therapies for myasthenia gravis. The study by Fang et al. investigated telitacicept, a recombinant fusion protein that targets the BAFF (B-cell activation factor)/APRIL (a proliferation-inducing ligand) pathway, as a treatment for MG. This study is timely because telitacicept targets BAFF/APRIL upstream in the immune cascade, potentially modifying disease by curbing the generation and survival of antibody-secreting cells rather than only removing circulating IgG. The findings contrast with mixed randomized results for rituximab in AChR-positive MG and the negative gMG trial of BAFF-only inhibition with belimumab, suggesting dual BAFF/APRIL blockade may overcome limitations of CD20 and BAFF-only strategies. By acting earlier in B-cell biology, telitacicept could offer more durable control and steroid-sparing benefits consistent with a disease-modifying profile. However, the follow-up was relatively short and the number of subjects small, so confirmation in larger, longer, randomized studies is needed. Zhang Z. et al. demonstrated safety and efficacy of efgartigimod (EFG), a neonatal Fc receptor antagonist, as a fast-acting treatment option for 15 patients with generalized very-late-onset myasthenia gravis (VLOMG). Specifically, the study reported a clinically meaningful improvement rate in most of the overall cohort, with a significant change in MG-ADL scores. The authors highlight the potential for EFG as a rapidly effective intervention with sustained clinical benefits in this older and often more fragile patient population. Additionally, this strategy also facilitates prednisone dose reductions, helping achieve disease control with fewer corticosteroid-related side effects, a common concern in this group. These studies collectively enhance our understanding of newer treatment targets and strategies for MG.

Finally, the review article by Groener and Paik provides a comprehensive synthesis of the rapidly evolving landscape of B lineage-directed therapies in idiopathic inflammatory myopathies (IIM). This review explores cutting-edge treatments—including next-generation anti-CD20s, BAFF inhibitors, CD19 CAR-T cells, BCMA-targeted approaches, and anti-CD38 antibodies—and frames a potential paradigm shift for addressing refractory disease. The review tackles a critical unmet need, drawing attention to the limited efficacy of existing biologics and the low-certainty evidence that has characterized IIM trials, despite the approval of IVIG and the widespread use of conventional immunosuppressants. Mechanistically, the article addresses why more precise targeting of plasmablasts and plasma cells could significantly impact disease outcomes. It links autoantibody biology to clinical phenotypes, suggesting that therapies like BCMA/CD38 strategies and CD19 CAR-T cells may hold promise for achieving more durable remissions. The translational relevance of these findings extends beyond myositis, to other immune-mediated neuromuscular disorders such as myasthenia gravis, as well as broader autoimmune diseases, where advancements in B-cell and plasma-cell therapies are already reshaping treatment paradigms. Importantly, the review emphasizes the need for larger, controlled clinical trials and thoughtful trial design in rare diseases to better define the long-term safety, efficacy, and optimal use of these therapies.

These articles, providing contributions ranging from insights into cellular mechanisms in MG to diagnostic approaches for IIM, highlighting the growing utility of autoantibody profiling in characterizing disease and informing therapeutic strategies. These promising insights would hopefully inspire future research aimed at enhancing patient outcomes in complex neuromuscular disorders aligned with the goal of personalized, precision care.
